# Thermal Behavior Modeling Based on BP Neural Network in Keras Framework for Motorized Machine Tool Spindles

**DOI:** 10.3390/ma15217782

**Published:** 2022-11-04

**Authors:** Aleksandar Kosarac, Robert Cep, Miroslav Trochta, Milos Knezev, Aleksandar Zivkovic, Cvijetin Mladjenovic, Aco Antic

**Affiliations:** 1 Faculty of Mechanical Engineering, University of East Sarajevo, 71123 Istocno Sarajevo, Bosnia and Herzegovina; 2 Faculty of Mechanical Engineering, VSB-Technical University of Ostrava, 70833 Ostrava, Czech Republic; 3 Faculty of Technical Sciences, University of Novi Sad, 21000 Novi Sad, Serbia

**Keywords:** high-speed motorized spindle, thermal behavior, deep learning, neural network, small dataset, Keras, TensorFlow

## Abstract

This paper presents the development and evaluation of neural network models using a small input–output dataset to predict the thermal behavior of a high-speed motorized spindles. Different neural multi-output regression models were developed and evaluated using Keras, one of the most popular deep learning frameworks at the moment. ANN was developed and evaluated considering the following: the influence of the topology (number of hidden layers and neurons within), the learning parameter, and validation techniques. The neural network was simulated using a dataset that was completely unknown to the network. The ANN model was used for analyzing the effect of working conditions on the thermal behavior of the motorized grinder spindle. The prediction accuracy of the ANN model for the spindle thermal behavior ranged from 95% to 98%. The results show that the ANN model with small datasets can accurately predict the temperature of the spindle under different working conditions. In addition, the analysis showed a very strong effect of type coolant on spindle unit temperature, particularly for intensive cooling with water.

## 1. Introduction

The importance of machine tool manufacturing in the metal machining industry is evident today, and it has a twofold effect on the industry. First, machine tool manufacturing as a part of the industry affects the position and the importance of the industry through its level of development and manufacturing output. Second, machine tool manufacturing creates the means of the working process for the metal-cutting industry, which, in turn, increases its total efficiency. In today’s prosperity of industrial rise, the application of the high-speed motorized spindle has significantly increased machining productivity and reduced manufacturing cost. However, its high speed and compact structure impose some negative effects on spindle thermal behaviors. Heat generation from spindle motors and bearings generally influence the temperature rise of a motorized spindle unit in its operation, resulting in errors, which are the main reasons for the reduction in machine tools’ precision and accuracy. The thermal behavior, caused by the temperature rise, is one of the main causes of the inaccuracy of the machine tools. Thermal errors account for 60 to 70% of total machine tool errors [1]. On the other hand, approximately 75% of machined workpieces’ geometrical errors occur due to temperature influence [2]. The heat generation on the bearings and motor occurring during the operation of high-speed spindles is often considerably high, so active cooling is required. With the correct choice of coolant and coolant flow, it is possible to influence the temperature rise and thereby reduce errors due to heat load. For active cooling of the housing, water with anticorrosive additive or special oil is used. The application of cooling oil is particularly popular in warm and humid Asian areas. This is due to the oil’s lower susceptibility to microbial contamination. Air and oil are also used as cooling liquids for the bearings. A large number of cooling strategies and concepts are available in [3] to reduce undesired effects due to thermal loads. On the other hand, challenges due to heat in motorized spindle units are currently being addressed by the industry and in research to improve the thermal properties of the spindle unit. At present, research on the thermal behavior of machine tools is being developed in several directions: numerical modeling, experimental testing, and model development based on artificial neural networks (ANNs). Compared with experimental measurement, numerical methods (using commercial softwaresuch as Ansys 12.0, MATLAB R16b, Abaqus2020, etc.) and ANNs allow comprehensive knowledge of the thermal behavior of the machine tools, and they save the time and energy cost of the experimental tests.

Many researchers have applied the finite element method (FEM) [4,5,6,7,8,9,10,11,12], the finite difference method (FDM) [13,14], or the finite difference element method (FDEM) [15,16,17] to obtain the numerical solution of temperature field of the spindle or of the entire machine tool. On the other hand, the temperature field of the spindle can be obtained by using the thermal network [18,19,20,21] or bond graph method [22], whereby, thermal balance equations can be solved by the Newton–Raphson method.

The above methods are limited due to the problems of defining the boundary conditions and the determination of the characteristics of heat transfer. In the recent decade, it has been shown that thermal behavior can be predicted by empirical modeling techniques, such as regression analysis, ANN, Grey system theory, and a combination of different modeling methods, with satisfactory accuracy. However, the temperature and the thermal displacement usually change with the operating conditions and the environment; it is difficult to apply regression analysis to a multiple-output-variable model, such as the model in this paper. Different from the regression model, the spindle thermal behavior in multiple directions can be modeled with only one neural network, as it has multiple outputs.

However, defining ANN models is not simple and depends firstly on the problem complexity and the learning algorithm complexity. Many researchers have used the trial-and-error method to improve the performance of neural networks. Through this process, neural networks are tested and evaluated, and then the optimal structure is determined. A method to select the number of hidden layers and nodes within, based on the numbers of input and output variables, was proposed in [23]. Bebis et al. [24] reported that small feed-forward neural networks can predict more accurately than larger ones. In addition, Kosarac et al. [25] showed that ANNs can be successfully trained with small data and used to predict the arithmetic mean roughness. In cases where the experiment is well planned and carried out, and if there is a high correlation between input and output, it is possible to achieve good neural models with small datasets, as presented in [26,27,28].

The most commonly used neural network methods are backpropagation (BP) and radial basis function (RBF). They have good performance in mapping and predicting the spindle thermal behavior. However, they often take a long time to train, and the speed of the convergence is slow. To overcome these drawbacks, researchers used different algorithms to optimize the structure of the BP neural network and avoid unstable prediction performance. A model based on the five key temperature points for the prediction of thermal deformation in the turning center by using a genetic algorithm (GA)-based BP neural network was proposed by Hao et al. [29]. Additionally, Huang et al. [29] introduced a genetic algorithm (GA) to optimize the BP network’s initial weights and thresholds. To improve the prediction performance of the neural network, Feng et. all [30] integrated multiple BP neural network models. Li et al. [31] performed optimization of the weights and thresholds of the BP neural network using the variable inertia factor particle swarm algorithm. In their paper, the temperature measurement points were clustered by the self-organizing feature map neural network. The correlation analysis method was used to explore the correlation between the thermal sensitive points and the thermal error of the spindle. Li et al. [32] used a beetle antenna search algorithm (BAS) to optimize the weights and thresholds of the BP neural network. The authors showed that BAS-BP had higher prediction accuracy than the BP and GA-BP models at different speeds. The RBF neural network is also a feed-forward (FF) network that has good approximation and global optimal performance. Cui et al. [33] proposed a model based on the five-point method using the multiple linear regression (MLR) method, BP, and RBF neural network to establish the thermal error prediction of the motorized spindle. Lv et al. [34] improved the prediction accuracy and generalization ability of ANNs by using a generalized RBF neural network modeling method and applied it to the thermal error modeling of the spindle housing. Zhang and Fu [35,36] improved the accuracy of the RBF neural network, developing the thermal error prediction model. They applied the genetic algorithm, particle swarm algorithm, and chicken flock algorithm to optimize the important parameters (hidden layer and output layer weights) of the RBF neural network. Dynamic neuron network models have better robustness in modeling spindle thermal behavior although temperatures are changing and the thermal–elastic process is varying nonlinearly under different working conditions [37]. Kang et al. [38] proposed a modified method that combined a feed-forward neural network (FNN) and hybrid filters for the prediction of thermal deformation in a machine tool. The hybrid filter consists of linear regression (LR), moving average (MA), and autoregression (AR). Outputs from the filter serve as input of the FF network, which is estimated by the static and dynamic relationships between the temperature distributions and thermal deformations.

In addition to those widely used neural network models discussed above, other neural networks were studied and applied for analysis of the spindle thermal behavior. Yang et al. [39] proposed a modified Elam network (EN) for determining the thermal errors of the spindle based on the FEA simulation. Li et al. [40] optimized weights and thresholds of the Elman neural network by using the sparrow search algorithm to predict thermal errors in motorized spindles. Zhang [41] proposed serial grey neural network (SGNN) and parallel grey neural network (PGNN) to predict the thermal error. Abdulshahed et al. [42] proposed a methodology for thermal error compensation using a grey neural network model with convolution integral optimized by a particle swarm optimization. Consideration of limitations and challenges in applying ML techniques along with possible strategies to address them, including computational cost, large errors of extrapolation, data availability, and the iteration with experiments, was presented by Qian and Yang [43]. Raza et al. [44] presented an unsupervised machine learning algorithm (including random forest, least absolute shrinkage, selection operator regression, and feed-forward neural networks) that can automatically classify and rationalize chemical trends in PFAS structures.

Thermal errors occur primarily due to temperature rise and temperature differences, which are the consequences of heat sources in the machine tool and changes in environmental conditions. Usually, the temperature variables tested by multiple sensors are taken as the input, and the thermal errors of machine tools are the output of NN model. The thermal and mechanical behavior of the spindle is a function of the cooling system. Inadequate coolants and flow lead to an increase in temperature and thermal expansion on the spindle elements. This causes an increase in thermal deformations as well as a decrease in the energy efficiency of the machine tool.

This paper presents the BP neural networks with the Adam optimization algorithm for the prediction of the temperature of motorized spindle units under different input conditions. The Adam optimization algorithm is implemented instead of the commonly used stochastic gradient descent (SGD) algorithm. Several models of neural networks have been developed where the number of hidden layers and neurons within varies. Spindle speed, coolant type, coolant flow of motor, and bearings were used as input parameters. Temperatures measured in three different areas of the spindle unit were considered as the output parameters. After the network learning and training, the temperatures of the motorized spindle unit in different areas were accurately fitted and predicted. The dataset used for the ANN development was obtained experimentally under various operating conditions. The experiment was conducted on the basis of the Box–Wilson central composite design with levels and ranges for three numerical (quantitative) factors and one categorical (qualitative) factor.

## 2. Experimental Setup

This paper investigates the effects of four factors on the temperature of a motorized spindle unit: a number of revolutions (*n*), coolant flow of motor (Q_m_), bearing coolant flow (Q_b_), and the coolant type (H). Table 1 shows these factors and their levels.

**Figure 1:**
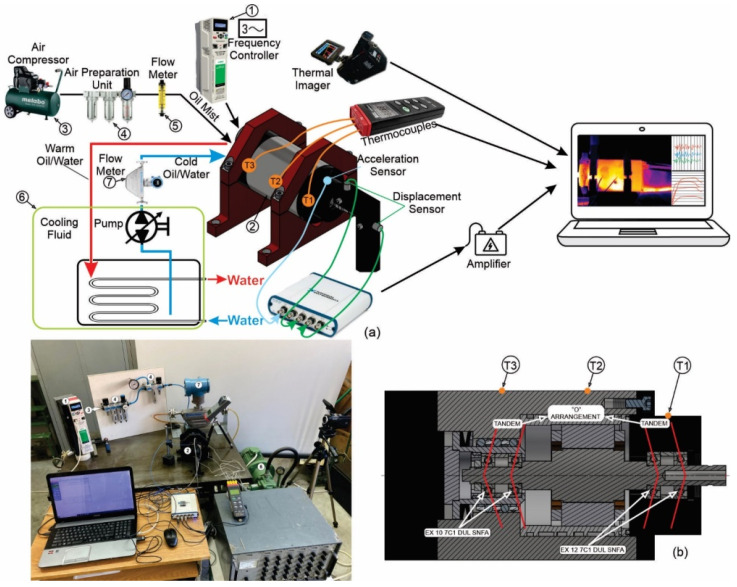
(**a**) A schematic of the experimental rig; (**b**) Spindle unit.

Figure 1a shows the experimental setup for measuring temperatures. Collected data were used to train and simulate neural networks. The tests were performed on a high-speed motorized spindle GMN TSSV 100-90000 (Figure 1b) with a maximum number of revolutions *n* = 90,000 [rpm], power P = 3 [kW], 2-pole AC induction electric motor, maximum U = 220 [V] per phase at maximum frequency. Cooling and oil–air lubrication were performed by injecting oil Q_b_ = 187.2–283.6 [mL/h] in all bearings simultaneously. Oil or water flowed through the groove around the stator with a flow rate of Q_m_ = 4–6 [L/min]. The spindle was mounted with two pairs of high-precision angular contact bearings, the front bearing with EX 12 7C1 DUL SNFA and the rear bearing with EX 10 7C1 DUL SNFA, mounted in a “tandem” arrangement so that the entire bearing formed an “O” arrangement. Front bearings had lock-ring preload, while rear bearings had constant (spring) preload. During the experiment, the external ambient temperature was 21 °C.

The motorized spindle (2) was connected to the frequency regulator Nidec HS 72 (1), which allowed for the desired RPM. The Acrylic Flow Meters 6A01 (5) was mounted to measure the amount of oil mist on the bearings at all times. The measurement of the flow of the cooling fluid for the motor was carried out with an Integral Flowmeter AXF (7) according to the principle of the Coriolis effect. The temperature of the cooling fluid was kept constant at 22 °C by using a heat exchanger placed in the tank (6). To measure the temperature of the spindle unit, thermocouples, infrared thermometer, and thermal imager were used. Three K-type thermocouples (T1, T2, and T3) were placed on the housing near the front and rear bearings and near the stator of the motor (Figure 1a,b). The temperature obtained from these thermocouples was collected by an acquisition system NI USB 6281 and then sent back to a computer for processing and monitoring results every one second. The output from the acquisition system was continuously monitored and analyzed in Matlab R16b software. An infrared thermometer was used to record the temperature at the outlet coolant from the spindle unit. To record the distribution of temperature fields, monitor the temperatures of the entire experimental rig, as well as to control the thermocouples and infrared thermometer, the Thermo Pro^TP^ TP8S, Wuhan Guide Infrared Co., Ltd., Wuhan, China, thermal imager was used. Table 2 shows the characteristics used for equipment.

### 2.1. Experimental Plan

The goal of this experiment was to determine the temperature rise on the spindle unit for different operation conditions. Using the Box–Wilson central composite design, the experiments were reduced to 40 runs. The central composite matrix shown in Appendix A (Table A1) contains 40 rows, representing the number of experimental runs.

In this research, levels and ranges were applied for three numerical (quantitative) factors: RPM, coolant flow, and oil mist flow. In addition, a categorical (qualitative) factor was applied—it was a type of cooling where oil and water were used. For a centrally composite plan, the parameter α, the distance of the axial arrays from the projected center, was 1, so that each numerical factor had three levels. The experiment was divided into two blocks. The role of the blocks was to reduce or eliminate the variability caused by interference factors that may have affected the response but were not directly related to a design factor. For this experiment, six replications were conducted to the midpoint for each level of the categorical factor, that is, two replicates for each level of the categorical factor in each block. For the central point in the project, standard working conditions were applied. Columns 8, 9, and 10 in Appendix A (Table A1) show the results of the measured temperatures (responses) for the conducted experiments in all three points, i.e., near front bearing, stator, and near rear bearing.

### 2.2. Analysis of Experimental Data

**Figure 2:**
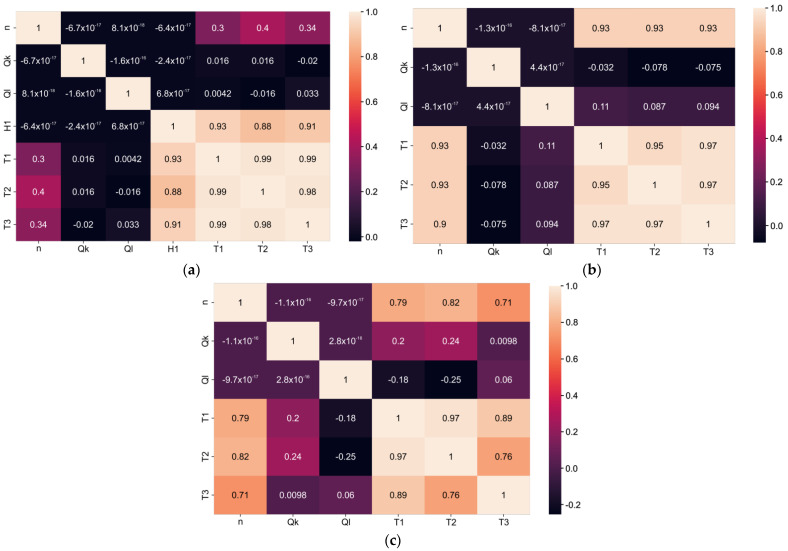
Correlation matrix; (**a**) four input variables; (**b**) coolant type oil; and (**c**) coolant type water.

Data analysis was performed using the Seaborn, Scikit-learn, NumPy, and Pandas machine learning libraries and Keras framework for Python. Various algorithms were used for data visualization, such as HeatMap or Scatterplot. Since string values in column H (Appendix A Table A1) are not appropriate for data analysis, values in the column were converted into Boolean using the replace method from the Pandas library. Linear relationships between variables could be shown and quantified with a correlation matrix. The correlation matrix is a square matrix that measures the linear dependence between pairs of attributes. The correlation coefficients range from −1 to 1, where two attributes have a perfect positive correlation if r = 1, no correlation if r = 0, and a perfect negative correlation if r = −1. On the basis of the above analysis, the comprehensive correlation between the temperature and input variables was obtained, as shown in Figure 2. Figure 2a shows a correlation matrix with a correlation coefficient for all input variables. The correlations between output variables and input variable “H” (coolant type) were 0.93, 0.888, and 0.91, indicating that they were strongly positively correlated (Figure 2a). The correlations between output variables and input variable *n* (spindle speed) were positive and had values 0.3, 0.4, and 0.34. That indicates that correlation existed but was not significant, as in the previous case. The correlations between output variables and input variables Q_m_ and Q_b_ (black highlighted cell in the correlation matrix) were negligible, indicating that this relationship was not noticeable. The correlations between output variables T1, T2, and T3 were positive and had values of 0.98 and 0.99. It can be seen from Figure 2a that the type of coolant had the biggest influence on motor-spindle temperatures. When water-cooling of the housing was used, a large amount of heat was transferred by water. Water-based cooling was generally more effective due to higher specific heat capacity (41.2 kJ/(kg·K) at 20 °C for water vs. 1.9 kJ/(kg·K) for special cooling oil).

If the correlation matrix is observed separately for the case of cooling with oils and cooling with water, the influence of certain input variables changes significantly. The correlation between input and output variables for oil cooling was positive and had a value of 0.93 (Figure 2b). The small correlation factor between oil flow and temperature indicates that the oil flow did not significantly affect the spindle unit temperature change.

For the case of water-cooling, correlations between output variables and input variable *n* (spindle speed) were positive and had values of 0.79, 0.82, and 0.71. Correlations between Q_m_ flow, as an input variable, and output variables had values of 0.2, 0.24, and 0.098, which indicates that correlation was not high but existed (Figure 2c). Therefore, the water flow had an impact on the temperature change of the spindle unit.

## 3. BP Neural Network Modeling

This paper also considered the influence of the network topology and the learning parameter on the model performances. Several models of neural networks have been developed where the number of hidden layers and neurons within varies. Neural network models have one, two, or three hidden layers. The number of neurons in the hidden layer varies from 2 to 10.

The Adam optimization algorithm is used for updating network weights in training data instead of the commonly used SGD algorithm. Adam algorithm uses two components, momentum and adaptive learning rate, to converge faster and update network weights efficiently. Momentum update can be expressed mathematically:

(1) $$\nu_{t}=\gamma \cdot \nu_{t-1}+\eta \cdot \nabla \cdot \theta \cdot J\left(\theta \right)\;\theta =\theta -\nu_{t}$$

In the above equation, *θ* is network parameter (weight, bias…), *η* is a learning rate, *J* is a function to optimize, *γ* is a constant, *ν*_(*t*−1)_ is a past timestep, and *ν_t_* is a current timestep.

Adaptive learning rates can be observed as adjustments, i.e., reducing the learning rate in the training phase. Mathematical expression is given in following equation:

(2) $$E{\left|g^{2}\right|}_{t}=\beta E{\left|g^{2}\;\right|}_{t-1}+\left(1-\beta \right)g_{t}^{2}\;\Theta_{t+1}=\Theta_{t}-\frac{\eta }{\sqrt{E{\left|g^{2}\;\right|}_{t}+\varepsilon }}\cdot g_{t}$$

where $E{\left|g^{2}\right|}_{t}$ is an exponentially decaying average of squared gradients, *β* has the recommended setting of 0.9, *θ*_*t*+1_ denotes the resulting (updated) parameters, and *g_t_* is the gradient at timestep *t*.

### 3.1. Impact of Network Architecture on Model Performance

The activation function for hidden layers is ReLU, and the linear activation function is used for the output layer. The number of epochs is set at 10,000, but the early stopping function interrupts training after model performance stop to improve on the validation dataset. First, for model selection and evaluation, the hold-out method is used. R-squared and RMSE are used to explain a quantitative measure of model performances for each output, and the average value is considered.

Table 3 shows that network performances are not improving significantly by increasing the number of hidden layers and neurons within. A smaller value of the learning parameter improves the performance of a neural network that has more than one hidden layer but slows down the training at the same time; however, it should certainly take into account that the difference in the results of individual networks is negligibly small. It is important to note that in this case, the dataset is divided randomly, which means that during the next training, the results will likely differ from the results shown in Table 3.

### 3.2. Impact of Cross-Validation Technique on Model Performance

Building the machine learning model requires two things: feeding the model with an initial dataset for training and then providing unseen data to the model to evaluate its performance. The stability and performances of the model highly depend on the data belonging to training and validation sets. Although a large number of evaluation techniques exist, this paper examines and compares the impact of two cross-validation techniques on model performance:

1. Holdout cross-validation technique
2. KFold cross-validation technique.

**Figure 3:**
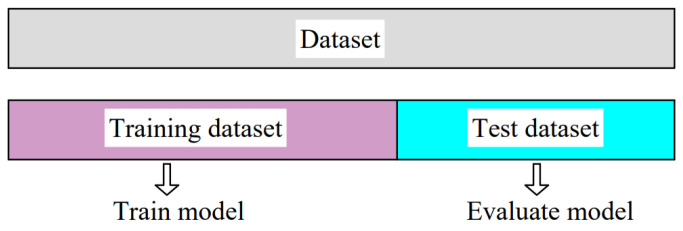
Holdout cross-validation.

Holdout (Figure 3) is the simplest of all validation techniques widely used in machine learning. Holdout implies that the entire data set is divided, most often randomly, into two sets. Usually, data are divided in a ratio of 2/3 of the data to the training set and 1/3 of the data to the test set. If handling a large dataset, data can be divided into ratios of 60/40, 70/30, 80/20, or even 90/10. An advantage of this technique is that it is fast because training executes only once. As a drawback, randomly splitting the initial dataset can lead to high variance on repeated training. As a result, the model accuracy will not be consistent. The way to obtain a more robust model using the holdout method is by repeating training several times, using different random seeds. After k repetitions, average performance is computed.

**Figure 4:**
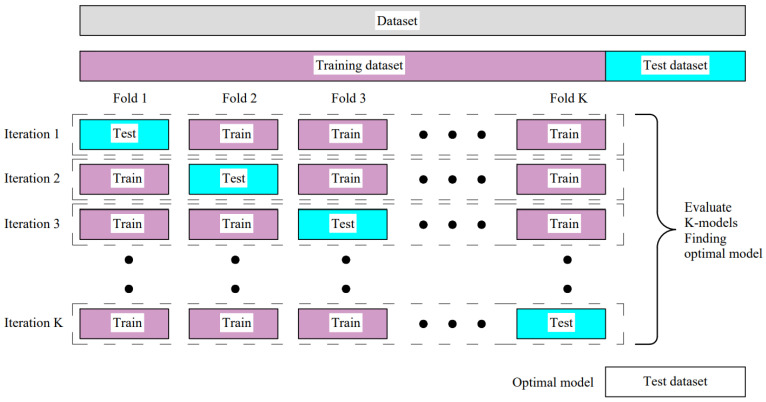
KFold cross-validation.

KFold cross-validation (Figure 4) is another method for estimating the model performances. This method systematically creates and evaluates multiple models on different subsets. In most cases, cross-validation is used for the estimation and evaluation of the effectiveness of different hyperparameters. The KFold validation technique is not desirable in neural networks because it is more expensive compared with the holdout technique (i.e., it is time-consuming). When the dataset is large enough, one validation set is enough, and usually there is no need for the KFold.

### 3.3. Choosing the Number of Folds for Cross-Validation

This section of the research analyzes the influence of the fold size on the model performance. Fold sizes from k = 2 to k = *n* were used to assess and evaluate their effects on prediction errors. The last case is a leave-one-out-cross-validation technique or LOOCV. LOOCV represents the cross-validation case where there are several folds, which are equal to dataset size, and just one fold is held out for validation.

In this research, the dataset with *n* = 40 input/output sets was split into folds. The same random-state function values (42), provide the same division into a training and testing set in all runs. Five different network architectures, 4-2-3, 4-4-4, 4-4-4-3, 4-8-8-3, and 4-10-10-3, were assessed.

**Figure 5:**
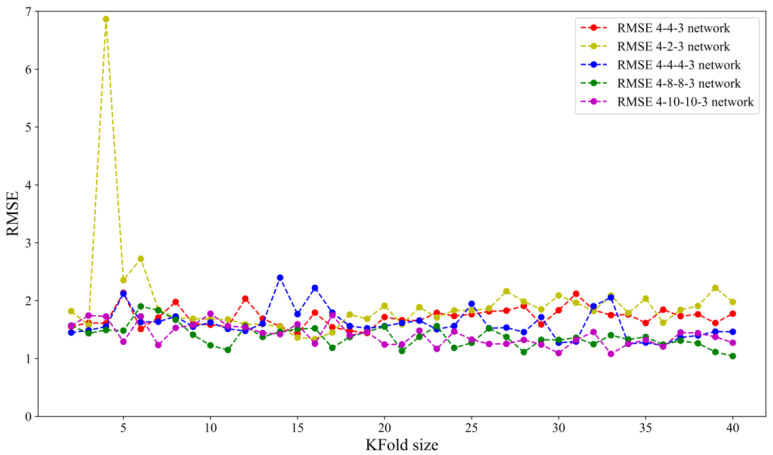
RMSE vs. KFold.

**Figure 6:**
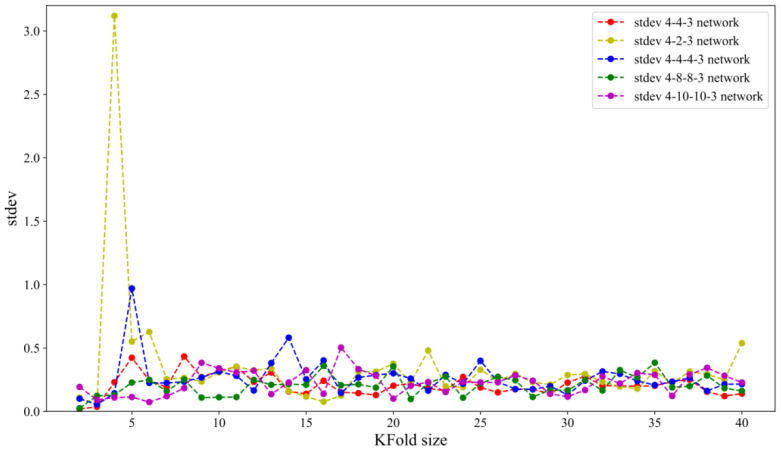
Standard deviation of RMSE vs. KFold.

The most common measures of regression model fit, R-squared and RMSE were used to estimate model performances. Root mean squared error can be expressed by formula:

(3) $$RMSE=\sqrt{\frac{1}{n}\sum_{i=1}^{n}{\left(y_{i}-\hat{y_{i}}\right)}^{2}}$$

where ${\hat{y}}_{i}$ is predicted values, and *y_i_* is a dependent regression variable. Since the training set has 40 samples, the 2-fold cross-validation estimates the model performance over a training set of 20, 4-fold cross-validation over a training set of 30, and so on, while LOOCV estimates the model performance over a training set of 39. Figure 5 and Figure 6 show the impact of KFold size on bias and variance for five tested network architectures.

Figure 5 shows that leave-one-out cross-validation does not lead to significantly lower bias than KFold. Increasing K slightly improves variance for almost all tested network architectures (except the 4-2-3 network architecture), as can be seen in Figure 6. This can be explained in the following way: for smaller *K* values, training samples become smaller. As a result, the model is less stable, which is indicated by the higher value of the variance.

**Figure 7:**
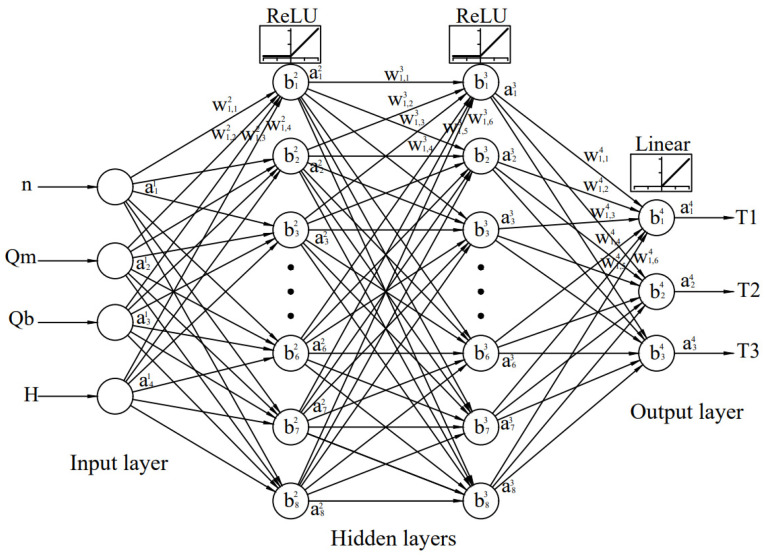
The adopted neural network topology 4-8-8-3.

Table 4 shows that the lowest average bias and variance has neural network topology 4-8-8-3 and it will be considered the most favorable. The topology of the adopted neural network for further analysis is shown in Figure 7.

## 4. Results and Discussion

**Figure 8:**
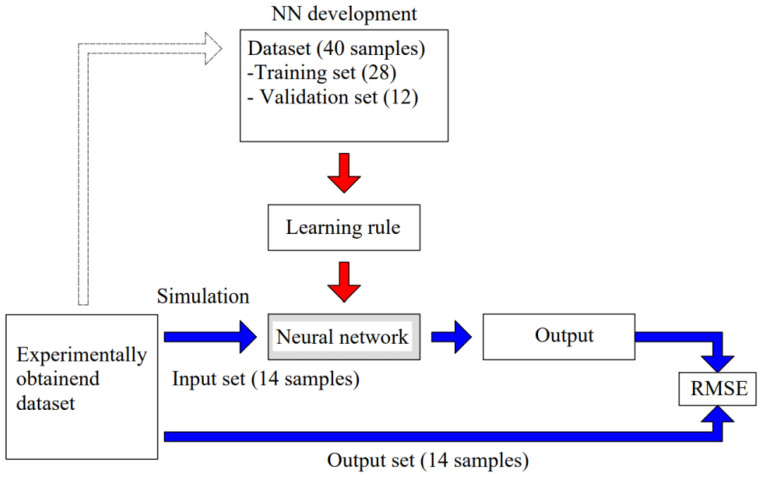
Simulation of the neural network using experimentally obtained dataset and developed NN model.

Figure 8 (based on [25]) shows the procedure of neural network building and simulation using an experimentally obtained dataset. In total, 40 samples were used for neural network model building, of which 28 samples were used for training and 12 for validation of the network. Following that, the neural network was simulated using 14 sets which were completely unknown to the network.

### 4.1. Verification of ANN Model

Network characteristics have been estimated based on the value of the root mean square error (RMSE) related to the simulation dataset and correlation coefficient (R). After comparing the absolute error of the experimentally obtained output and simulation output, an assessment of the possibility of using neural networks trained with small data sets was produced (Table 5).

The relative error was calculated by the equation:

(4) $$err.=\frac{T_{iNN}-T_{iexp}}{T_{iexp}}\times 100\%$$

**Figure 9:**
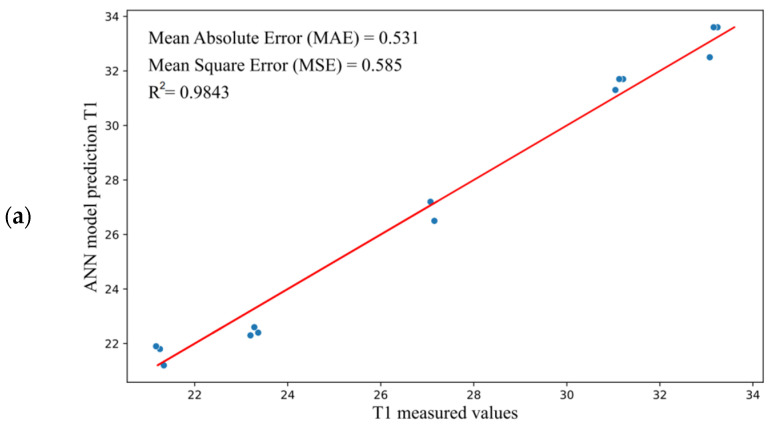

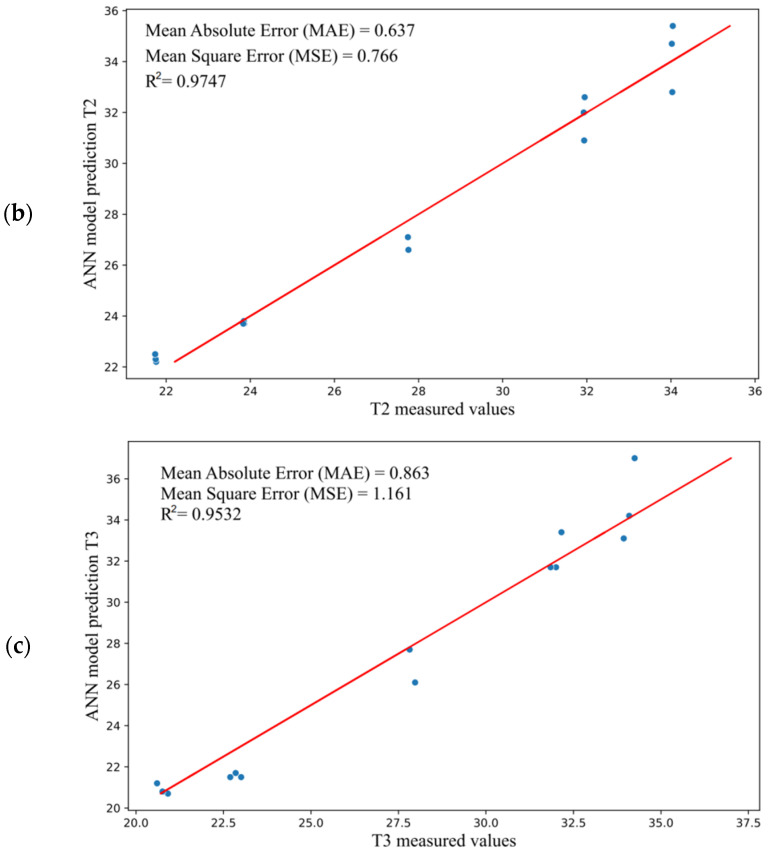
The plot of data regression for (**a**) temperature T1, (**b**) temperature T2, and (**c**) temperature T3.

The regression plot for simulation is shown in Figure 9. Figure 9a–c show that the neural network model provided R-squared values of R^2^ = 0.9843 (output T1—Figure 9a), R^2^ = 0.9747 (output T2—Figure 9b), and R^2^ = 0.9532 (output T3—Figure 9c), which can be considered good for all outputs.

### 4.2. Analysis of Working Conditions Effect on the Spindle Thermal Characteristics Obtained by the Proposed ANN Model

**Figure 10:**
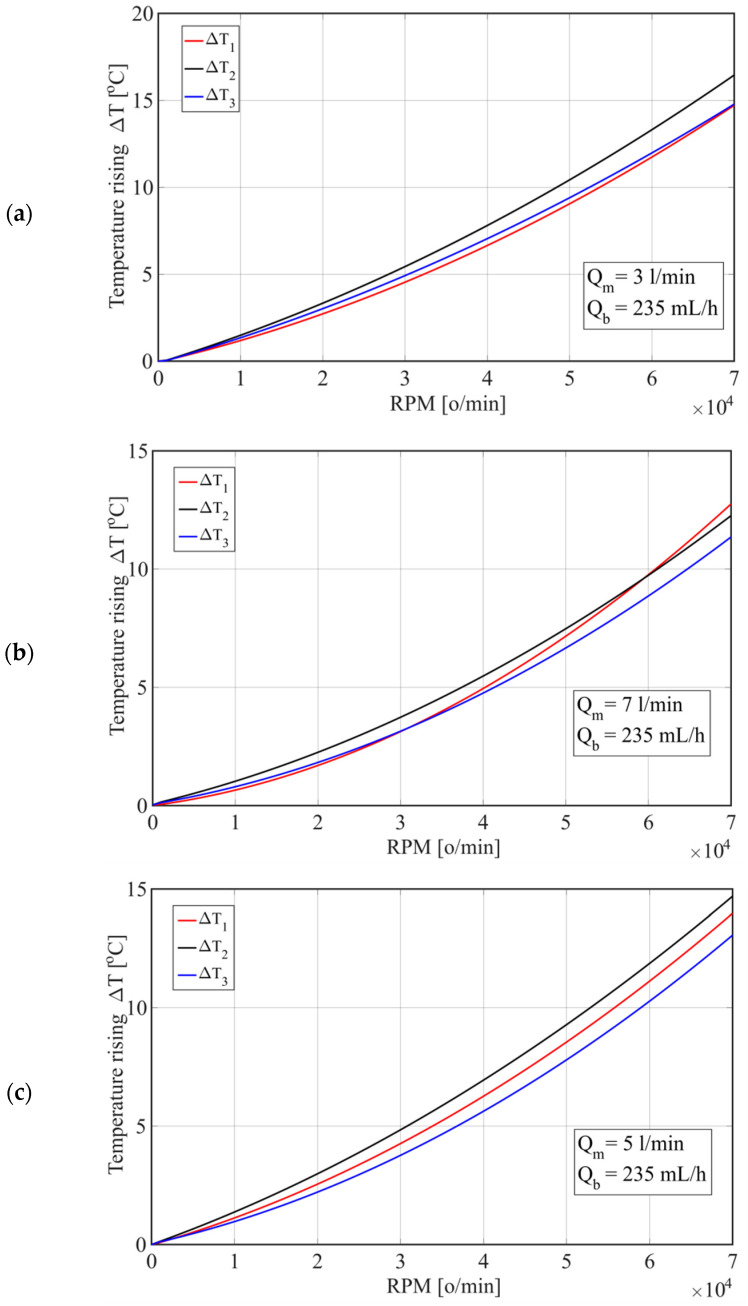
Temperature change depending on the number of revolutions for different oil flows through the housing: (**a**) Q_m_ = 3 [L/min]; (**b**) Q_m_ = 5 [L/min]; and (**c**) Q_m_ = 7 [L/min].

Figure 10 shows the change in temperature rise depending on the number of revolutions for different oil flows (Q_m_) through the housing. By increasing the number of revolutions by 25%, the temperatures of the spindle unit increased by 63% at an oil flow of Q_m_ = 3 L/min, i.e., by 65% at an oil flow of 7 L/min. However, by increasing the flow from Q_m_ = 3 L/min to Q_m_ = 7 L/min, the temperatures of T1 and T3 decreased by 2% at *n* = 30,000 rpm, or by 1% at *n* = 70,000 rpm, while the temperature of T2 decreased by 1.7% at *n* = 30,000 rpm and 3% at *n* = 70,000 rpm.

**Figure 11:**
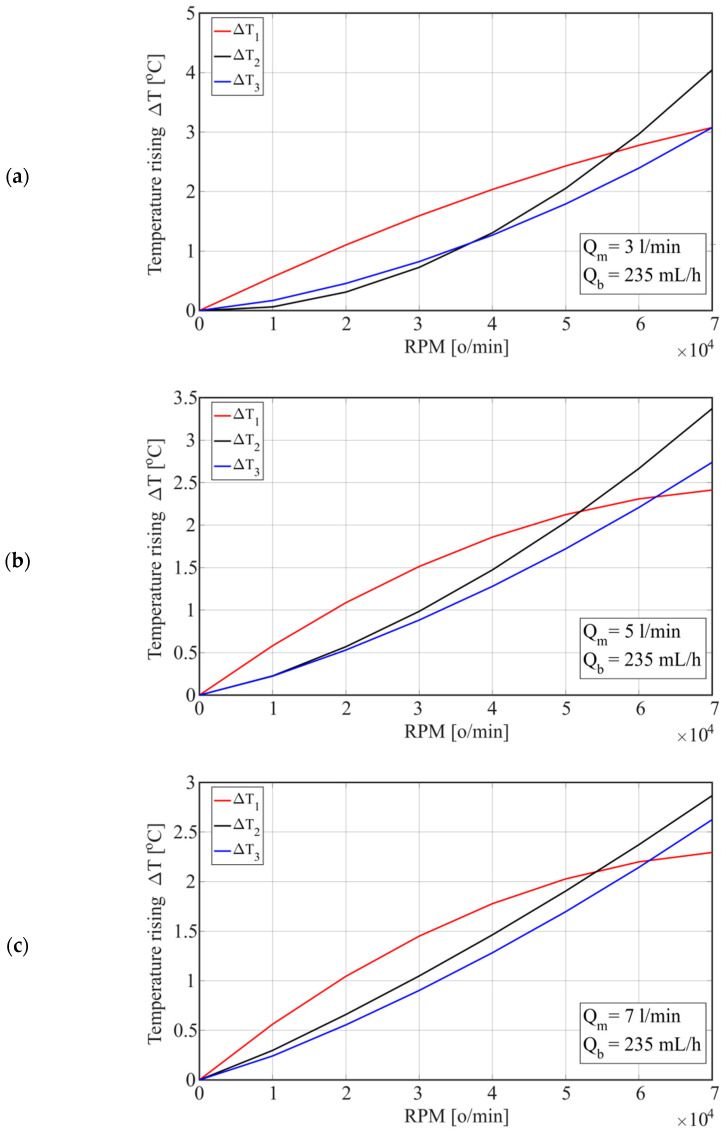
Temperature change depending on the number of revolutions for different water flows through the housing: (**a**) Q_m_ = 3 [L/min]; (**b**) Q_m_ = 5 [L/min]; and (**c**) Q_m_ = 7 [L/min].

The temperature change depending on the number of revolutions for the considered water flow (Q_m_) through the housing is shown in Figure 11. Increasing the number of revolutions from *n* = 30,000 rpm to *n* = 40,000 rpm (25%) increased the temperature difference (ΔT) at the considered points by 50% at a water flow of Q_m_ = 3 L/min, i.e., by 55% at a water flow of Q_m_ = 7 L/min. However, increasing the flow from 3 L/min to 7 L/min reduced the temperatures of T1 and T3 by 12% at *n* = 30,000 rpm, or by 13% at *n* = 70,000 rpm, while the temperature of T2 decreased by 10% at *n* = 30,000 rpm and 16% at *n* = 70,000 rpm.

When cooling the housing with oil, the maximum temperature increase ΔT2 was approximately 16 °C at a flow rate of Q_m_ = 3 L/min. By increasing the flow to Q_m_ = 7 L/min, ΔT2 decreased to 1 °C, while ΔT1 decreased from 14.5 °C to 13 °C, and ΔT3 decreased from 14.6 °C to 14 °C. On the other hand, when cooling the housing with water, by increasing the flow from 3 L/min to 7 L/min, the temperature ΔT1 decreased for 4 [°C], with a simultaneous decrease in the temperature ΔT2 from 3.8 °C to 1.2 °C and temperature ΔT3 from 3.9 to 1.3 °C. Therefore, the coolant flow had a greater effect on the temperatures of the spindle unit elements when cooling the housing (stator) with water, which is similar to the analysis of experimental test results presented in Section 2. With water cooling, the temperature ΔT2 was lower by approximately 12 °C than with oil cooling, while the temperatures ΔT1 and ΔT2 were lower by approximately 11 °C, which is also consistent with the experimental testing.

## 5. Conclusions

The major advantage of this approach is that it enables the results of ANN models to be easily integrated with FEM models or digital twins, especially for any further analyses of the influence of temperature on the spindle unit behavior.

Eighteen different neural network models were evaluated, whereby a hold-out method is used for model selection and evaluation. It can be seen that:

- Network performances does not improve significantly by increasing the number of hidden layers and neurons within;
- A smaller value of the learning parameter improves the performance of a neural network with more than one hidden layer but significantly slows down training;
- All models have high variance since the dataset was split randomly.

Five different neural network models were estimated using the KFold cross-validation technique.

- It can be seen that the model with the 4-8-8-3 network topology has the lowest RMSE value, which indicates that this model has the best performance.
- Increasing fold number has no significant impact on the bias but slightly improves variance.

The hold-out method is preferred when the dataset is large and can be a good validator for building the initial model. This method takes less computational power and requires less time to run.

- When handling small datasets, cross-validation is more desirable than the hold-out method since the model is trained on multiple folds. This provides a more reliable indicator of the model acting on unseen data. For datasets up to 40 input/output values, with multiple outputs, the LOOCV method provides the lowest bias and variance, and this model can be considered more reliable than models using lower Kfold values.

Through these findings, the history of temperature distribution on the spindle can be learned, and suitable coolant and flow for the motorized spindle unit can be chosen to minimize temperature rising and thermal expansion. The results of temperature obtained by means of the ANN model make it possible to indicate the best solution and to quantitatively assess the improvement in the high-speed motorized spindle thermal properties. By choosing the appropriate coolant and flow rate, the energy efficiency of the machine tool is increased, while temperature and errors due to heat load are reduced.

## Figures and Tables

**Table 1:** Experimental factors and their levels.

Condition	Factor	Units	Low Level	Middle Level	High Level
			−1	0	+1
Number of revolutions	*n*	o/min	40,000	55,000	70,000
Coolant flow of motor	Q_m_	L/min	4	5	6
Bearing coolant flow	Q_b_	mL/h	187.2	235.2	283.6
Coolant type	H	-	Oil		Water

**Table 2:** Main characteristic used measurement equipment.

	Thermocouples Type K	Thermal Imager	Acrylic Flow Meters	Integral Flowmeter AXF ^ISO 9104^	Infrared Thermometer
Operation range	−200 to 1200 °C	−20 to 250 °C	to 7 bar	to 100 bar	−40 to 500 °C
Accuracy	±0.2 °C at 100 °C	±1%	±5% from whole range	±0.15% from flow velocity	±0.2%

**Table 3:** Network topology and learning rate selected based on model performance.

	NN Architecture	No. of Epochs	Loss of Training Set	Loss of Evaluation Set	R2 (T1)	R2 (T2)	R2 (T3)	R2 (Aver.)	Learning Rate
					RMSE	RMSE	RMSE	RMSE (Aver.)	
1.	4-2-3	5059	1.731	2.404	0.892	0.835	0.905	0.877	0.01
					1.437	1.723	1.474	1.544	
2.	4-2-3	11,689	1.738	2.399	0.896	0.826	0.910	0.877	0.001
					1.418	1.767	1.436	1.540	
3.	4-3-3	2837	1.682	2.414	0.879	0.832	0.911	0.874	0.01
					1.521	1.705	1.421	1.549	
4.	4-3-3	8216	1.852	5.105	0.759	0.643	0.825	0.742	0.001
					2.195	2.479	2.084	2.252	
5.	4-4-3	3801	1.731	2.381	0.890	0.816	0.917	0.874	0.01
					1.439	1.784	1.374	1.532	
6.	4-4-3	9473	1.720	2.446	0.887	0.816	0.916	0.837	0.001
					1.472	1.801	1.388	1.553	
7.	4-5-3	1754	1.746	2.433	0.866	0.828	0.924	0.872	0.01
					1.606	1.722	1.323	1.550	
8.	4-5-3	8506	1.685	2.459	0.879	0.816	0.917	0.870	0.001
					1.520	1.777	1.380	1.559	
9.	4-3-3-3	118	7.979	24.12	0.025	−0.26	0.35	0.041	0.01
					5.141	5.045	4.527	4.904	
10.	4-3-3-3	4320	1.612	2.535	0.868	0.803	0.937	0.869	0.001
					1.626	1.879	1.195	1.566	
11.	4-4-4-3	124	13.992	26.011	0.146	−0.06	0.378	0.154	0.01
					5.198	5.196	4.899	5.097	
12.	4-4-4-3	7651	1.7867	2.3945	0.881	0.827	0.921	0.876	0.001
					1.523	1.752	1.338	1.537	
13.	4-8-8-3	93	13.7252	18.358	0.4685	0.0893	0.5202	0.359	0.01
					4.016	4.692	4.115	4.274	
14.	4-8-8-3	5214	1.7200	2.391	0.892	0.8312	0.9085	0.877	0.001
					1.445	1.728	1.448	1.540	
15.	4-4-4-4-3	590	1.084	3.381	0.842	0.757	0.915	0.838	0.01
					1.900	2.073	1.494	1.822	
16.	4-4-4-4-3	10,370	1.733	2.392	0.893	0.834	0.906	0.877	0.001
					1.429	1.723	1.471	1.541	
17.	4-4-10-4-3	4955	36.101	19.723	0	2.22	0	0.74	0.01
					4.35	4.16	4.788	4.43	
18.	4-4-10-4-3	6017	1.8126	2.4341	0.877	0.809	0.929	0.871	0.001
					1.533	1.828	1.268	1.543	

**Table 4:** Average values of RMSE and standard deviation for five network topologies.

Network Topology	RMSE	σ
4-2-3	1.9545	0.34766
4-4-3	1.7158	0.20772
4-4-4-3	1.6068	0.26232
4-8-8-3	1.3850	0.20518
4-10-10-3	1.4067	0.22730

**Table 5:** Comparison of experimentally obtained dataset and neural network predictions.

n	Q_m_	Q_b_	H	T1			T2			T3		
				Pred.	Measured	Err %	Pred.	Measured	Err %	Pred.	Measured	Err %
60,000	4	235.2	Oil	33.240	33.6	−1.07	34.044	35.4	−3.83	34.248	37.0	−7.44
60,000	5	235.2	Oil	33.156	33.6	−1.32	34.032	32.8	3.76	34.093	34.2	−0.31
60,000	6	235.2	Oil	33.073	32.50	1.76	34.021	34.70	−1.96	33.937	33.10	2.53
50,000	4	235.2	Oil	31.210	31.7	−1.55	31.949	32.6	−2.00	32.157	33.4	−3.72
50,000	5	235.2	Oil	31.127	31.7	−1.81	31.937	30.9	3.36	32.002	31.7	0.95
50,000	6	235.2	Oil	31.044	31.30	−0.82	31.926	32.00	−0.23	31.846	31.70	0.46
60,000	4	235.2	Water	23.365	22.4	4.31	23.856	23.7	0.66	23.004	21.5	7.00
60,000	5	235.2	Water	23.282	22.60	3.02	23.845	23.80	0.19%	22.848	21.70	5.29
60,000	6	235.2	Water	23.199	22.30	4.03	23.833	23.70	0.56	22.693	21.50	5.55
50,000	4	235.2	Water	21.337	21.2	0.65	21.761	22.2	−1.98	20.912	20.7	1.02
50,000	5	235.2	Water	21.253	21.80	−2.51	21.750	22.30	−2.47	20.757	20.80	−0.21
50,000	6	235.2	Water	21.170	21.90	−3.33	21.738	22.50	−3.39	20.602	21.20	−2.82
30,000	4	235.2	Oil	27.153	26.5	2.46	27.759	26.6	4.36	27.975	26.1	7.18
30,000	5	235,2	Oil	27.069	27.2	−0.48	27.747	27.1	2.39	27.819	27.7	0.43

## Data Availability

The data that support the findings of this study are available from the corresponding author upon reasonable request.

## Appendix A

**Table A1:** Experimental plan.

Randomized Row	No.	Block	*n* [rpm]	Q_m_ [L/min]	Q_b_ [mL/h]	H	T1 [°C]	T2 [°C]	T3 [°C]
	1	2	55,000	6	235.2	Oil	31.3	32.0	31.7
27	2	2	55,000	4	235.2	Oil	30.9	32.7	33.5
34	3	2	70,000	5	235.2	Water	23.5	25.3	22.5
31	4	2	55,000	5	235.2	Oil	31.9	31.3	31.8
39	5	2	55,000	5	235.2	Water	21.8	22.4	20.9
37	6	2	55,000	5	187.2	Water	21.7	22.5	20.8
26	7	2	70,000	5	235.2	Oil	34.5	38.1	35.9
40	8	2	55,000	5	235.2	Water	21.8	22.4	20.9
25	9	2	40,000	5	235.2	Oil	29.8	28.8	29.0
35	10	2	55,000	4	235.2	Water	22.0	22.6	21.2
32	11	2	55,000	5	235.2	Oil	31.9	31.3	31.8
38	12	2	55,000	5	283.6	Water	21.2	22.0	20.4
33	13	2	40,000	5	235.2	Water	20.7	20.6	19.8
36	14	2	55,000	6	235.2	Water	21.9	22.5	21.2
30	15	2	55,000	5	283.6	Oil	31.9	32.4	33.1
29	16	2	55,000	5	187.2	Oil	29.4	30.1	30.2
3	17	1	40,000	6	187.2	Oil	28.3	28.3	28.1
16	18	1	70,000	6	187.2	Water	25.8	27.6	24.9
15	19	1	40,000	6	187.2	Water	22.2	22.7	21.5
19	20	1	40,000	6	283.6	Water	21.8	21.9	21.2
5	21	1	40,000	4	283.6	Oil	30.3	30.3	30.1
17	22	1	40,000	4	283.6	Water	21.4	21.2	21.6
4	23	1	70,000	6	187.2	Oil	36.4	37.1	38.8
8	24	1	70,000	6	283.6	Oil	35.8	36.6	38.3
20	25	1	70,000	6	283.6	Water	24.1	25.9	23.2
10	26	1	55,000	5	235.2	Oil	31.9	31.3	31.8
13	27	1	40,000	4	187.2	Water	21.3	21.7	20.5
1	28	1	40,000	4	187.2	Oil	29.3	29.0	28.9
6	29	1	70,000	4	283.6	Oil	35.8	37.2	38.5
24	30	1	55,000	5	235.2	Water	21.8	22.4	20.9
23	31	1	55,000	5	235.2	Water	21.8	22.4	20.9
12	32	1	55,000	5	235.2	Oil	31.9	31.3	31.8
11	33	1	55,000	5	235.2	Oil	31.9	31.3	31.8
22	34	1	55,000	5	235.2	Water	21.8	22.4	20.9
9	35	1	55,000	5	235.2	Oil	31.9	31.3	31.8
7	36	1	40,000	6	283.6	Oil	29.6	29.6	29.5
18	37	1	70,000	4	283.6	Water	23.5	23.3	25.5
2	38	1	70,000	4	187.2	Oil	36.2	37.8	39.0
21	39	1	55,000	5	235.2	Water	21.8	22.4	20.9
14	40	1	70,000	4	187.2	Water	24.2	26.0	23.0
